# Clinical Significance of Thyroid-Stimulating Hormone Receptor Gene Mutations and/or Sodium-Iodine Symporter Gene Overexpression in Indeterminate Thyroid Fine Needle Biopsies

**DOI:** 10.3389/fendo.2018.00566

**Published:** 2018-09-25

**Authors:** Haixia Guan, Danielle Matonis, Gianluca Toraldo, Stephanie L. Lee

**Affiliations:** ^1^Department of Endocrinology and Metabolism, The First Hospital of China Medical University, Shenyang, China; ^2^Section of Endocrinology, Diabetes and Nutrition, Boston Medical Center, Boston, MA, United States

**Keywords:** thyroid nodule, genetic diagnosis, fine needle biopsy, *TSHR*, *NIS*, genetic alterations

## Abstract

**Objectives:** To examine the prevalence of genetic alterations of thyroid-stimulating hormone receptor (*TSHR*) gene and sodium-iodine symporter (*NIS*) in a series of thyroid fine needle biopsy (FNB) specimens with indeterminate cytology, and to assess the correlation of the type of genetic changes with clinical features and follow-up results in the target thyroid nodule.

**Methods:** Between February 2015 and September 2017, 388 consecutive FNBs with indeterminate cytology were evaluated for *TSHR* mutations and *NIS* gene overexpression using ThyroSeqV.2 next-generation sequencing (NGS) panel. Medical records were reviewed for target nodules.

**Results:** Among 388 indeterminate FNBs, *TSHR* mutations and/or *NIS* overexpression were detected in 25 (6.4%) nodules. Ten nodules (2.6%) harbored *TSHR* mutations only, 7 nodules (1.8%) over-expressed *NIS* gene only, and 8 nodules (2.1%) had both alterations. The *TSHR* mutations were located between codons 281 and 640, with codon 453 being the most frequently affected. The allelic frequency of the mutated *TSHR* ranged from 6 to 36%. One nodule with *NIS* overexpression was simultaneously detected *EIF1AX* mutation and *GNAS* mutation. Nodules with *TSHR* mutations and/or *NIS* overexpression presented hyperfunctioning (*n* = 4), hypofunctioning (*n* = 5), and isofunctioning (*n* = 3) on the available thyroid scintigraphies. Eight cases accompanied with hyperthyroidism in which only 1 was caused by the target nodule. Evidence of co-existing autoimmune thyroid disease (AITD) and multinodular goiter were found in 52% and 52% of cases, respectively. Seven nodules underwent surgeries and all were benign on final pathology. None of 9 nodules with follow-up by ultrasound (3~33 mon, median 12 mon) showed grow in size.

**Conclusions:** *TSHR* mutations and/or *NIS* overexpression can be detected in pre-operative FNB specimens using the NGS approach. These genetic alterations occurred in 6.4% thyroid nodules in this consecutive series with indeterminate cytology. They present not only in hyperfunctioning nodules but also in hypo- or iso-functional nodules, indicating their prevalence may be higher than previously expected. Co-existing AITD was common in cases with these molecular alterations. None of our patients with *TSHR* mutations and/or *NIS* overexpression manifested malignant outcomes. How to use these two molecular markers in thyroid FNBs to guide our clinical practice warrants further investigation.

## Introduction

In the human thyroid, thyroid-stimulating hormone (TSH) activates both the cyclic adenosine monophosphate (cAMP) and the phospholipase C-diacylglycerol regulatory cascades. The former cascade positively controls the hormone-producing function and growth of thyrocytes, while the latter cascade contributes to the generation of the intracellular signals myoinositol-1.4.5-triphosphate (1,4,5-PIP3) and diacylglycerol, both of which act on thyrocytes to increase protein iodination and thyroid hormone synthesis (1–6). These fundamental roles of TSH in regulating thyroid growth and function are attained by binding to its receptor, TSH receptor (TSHR), and can be mimicked by activating mutations of the TSHR. Sodium-iodine symporter (NIS) is another known key protein of normal thyroid cell physiology. It is responsible for the active transport of iodide across the basolateral membrane of thyroid cell. NIS function results in a 20- to 40-fold elevation of iodide concentration with respect to the iodide level in circulating blood (7–9).

Given the critical roles of TSHR and NIS in maintaining normal thyroid cell function and proliferation, it is expected that alterations of genes coding these two proteins (*TSHR* and *NIS* genes) will be implicated, at least in part, in a variety of thyroid disorders including thyroid nodules In fact, since the 1990s, germline and somatic activating mutations of *TSHR* have been reported in familial nonautoimmune hyperthyroidism (FNAH), sporadic congenital nonautoimmune hyperthyroidism (SCNAH), and autonomously functioning thyroid nodules (AFTNs) (10); loss-of-function mutations of *TSHR* have been found to cause hypothyroidism and euthyroidism with elevated serum TSH (so-called “compensated hypothyroidism”) (11, 12). *TSHR* mutations have also been found occasionally in functional and rarely in non-functional malignant thyroid nodules (1, 13–21). It was suggested that a *TSHR* mutation concurrent with other thyroid cancer-related genetic alterations such as *BRAF, GNAS, RAS, TP53, TRK, PAX8*/*PPARr*, and *RET*/*PTC*, or *TSHR* mutations occurring with very high allelic frequency (allelic frequency >30%) may have a high probability of thyroid cancer (22–25). Similarly, markedly increased levels of *NIS* expression have been exhibited in active Graves' disease (GD) and AFTN (26). In contrast, defects of *NIS* expression exist in several cases of congenital hypothyroidism (27–29), and generally low levels of *NIS* expression is found in non-toxic multinodular goiter (NMNG), diffuse iodine deficiency goiter (IDG) (26), and solitary malignant thyroid nodules (30–32).

In view of these findings, it should be noted that *TSHR* mutations and *NIS* overexpression are closely associated with thyroid nodules, in particular with AFTNs. To our knowledge, so far studies of this kind are entirely performed using surgically resected tissues of thyroid nodules. In these studies, functional status and clinicopathological features of the nodule were already confirmed before performing a genetic examination. The major purpose of detecting alterations of *TSHR* and *NIS* in confirmed cases was to look for genetic etiologies of AFTNs. Despite available results, the clinical value of a *TSHR* mutation and/or *NIS* gene overexpression testing in all pre-operative indeterminate FNB specimens has not been studied.

For those cytologically indeterminate FNB specimens obtained from thyroid nodules, molecular testing has been recommended to assist the identification of thyroid cancer. Starting from February of 2015, we routinely use a large commercial multi-gene next generation sequencing (NGS) gene array to test all indeterminate FNB specimens. This array is called the ThyroSeqV.2 next-generation sequencing panel. With a high capability of sensitive detection, ThyroSeqV.2 can provide abundant information on a number of genes associated with thyroid tumor including *TSHR* and *NIS* in a given sample (33, 34). Utilization of ThyroSeqV.2 in all indeterminate cytology gives us an opportunity to find thyroid nodules harboring *TSHR* mutations and/or *NIS* overexpression. It allows us to investigate the clinical significance and diagnostic utility of *TSHR* mutations and/or *NIS* overexpression detected in pre-operative thyroid nodules with an indeterminate cytology.

Thus, aims of the present clinical utility study included examining the prevalence of genetic alterations of *TSHR* and *NIS* in a series of thyroid FNB specimens with indeterminate cytology, as well as identifying various forms of *TSHR* and *NIS* alterations. In addition, we intended to assess the correlation of the type of genetic changes with demographic features, sonographic patterns, functioning status, surgical pathology, and follow-up results in the target thyroid nodule.

## Subjects and methods

### FNB samples

Between February 2015 and September 2017, 1,293 consecutive FNBs were performed on thyroid nodules (>1 cm) under ultrasound (US) guidance (Toshiba Xario™ 200 with 5–14 MHz 58 mm Linear Array Ultrasound Transducer or 4.2–10.2 MHz micro convex or linear probe; Toshiba America Medical System, Tustin, CA) by endocrinologists at Boston Medical Center and interpreted by experienced cytopathologists. Cytology diagnosis was determined according to The Bethesda System for Reporting Thyroid Cytopathology (35). Among them, 388 FNB samples with indeterminate were sent consecutively to the CBL Path (Rye Brook, NY) for molecular analysis.

### Analysis of *TSHR* mutation and *NIS* overexpression

At the time of FNB aspiration or capillary technique, the content of the first FNB was preserved for molecular analysis, according to the manufacturer's instructions. *TSHR* mutations and *NIS* gene overexpression were detected using the ThyroSeqV.2 next-generation sequencing panel. This panel is applied to test for key thyroid cancer-related genes for base substitutions and small insertion/deletions in targeted regions of 14 genes (*AKT1, BRAF, CTNNB, EIF1AX, GNAS, NRAS, HRAS, KRAS, PIK3CA, PTEN, RET, TERT, TP53, TSHR*), for more than 42 types of gene fusions (involving *ALK, BRAF, IGF2BP3, MET, NTRK1, NTRK3, PPARG, RET, and THADA*), and for gene expression of *PGK, KRT7, TTF1, TG, CALCA, NIS (SLC5A5), PTH, KRT20* control genes. The analytical sensitivity for detection of all variant types including base substitution and insertion/deletions at >3–5% mutant allele frequency and gene fusions at >1% of tumor nuclei is >99.9% (95% CI: 98–100%). Gene expression profile is reported as negative (normal expression profile) or positive (abnormal expression of one or more genes) with detailed interpretation (33, 34).

### Medical review and follow-up of clinical characteristics of the biopsied nodules

A thorough review of the medical record was performed for each patient with a TSHR mutation and/or NIS overexpression. Patient demographic data, medical history, medical therapy, history of head and neck radiation, thyroid function tests (TFTs), thyroid autoantibody tests, thyroid ultrasound images, and ^123^I radioactive iodine thyroid scintigraphy scans (NM thyroid uptake and standard three-view planar scan) were collected and recorded. Cytology reports were reviewed to search for details in addition to Bethesda categories.

The biopsied nodules were clinically monitored by periodic bedside thyroid ultrasound performed by an endocrinologist to determine their clinical outcomes. For cases that underwent surgery after FNBs, histologic slides were reviewed by experienced pathologists. The surgical pathology of the thyroid nodule was used as the gold standard for indeterminate Bethesda cytology (Bethesda III, IV, and V) categories. For cases that underwent observation, periodical thyroid ultrasonography, TFTs, and radiological examinations were recommended. The associated reports were collected.

The target nodule was identified by correlating the site and size according to the associated reports with the pre-biopsied US images saved on the radiology picture archiving and communication system (PACS), to ensure that cytology, molecular analysis, radiology, and pathology reports belonged to the same specific nodule.

### Statistical analysis

Data were analyzed using SPSS software (SPSS 20.0, IBM, USA). The parametric variables were compared using the unpaired *t*-test between two groups and Kruskal–Wallis one-way ANOVA test among three groups. Difference between frequencies was compared using the Yates 2 × 2 Chi square test (when there were <10 numbers in data) or Fisher's test (when there were <4 numbers in data). *P* level of 0.05 or less was considered significant.

### Ethics

The protocol of this study was approved by the Boston University Institutional Review Board. Informed consent was obtained from all patients before performing the procedure.

## Results

### Genetic alterations of *TSHR* and *NIS* in indeterminate FNBs

**Figure 1:**
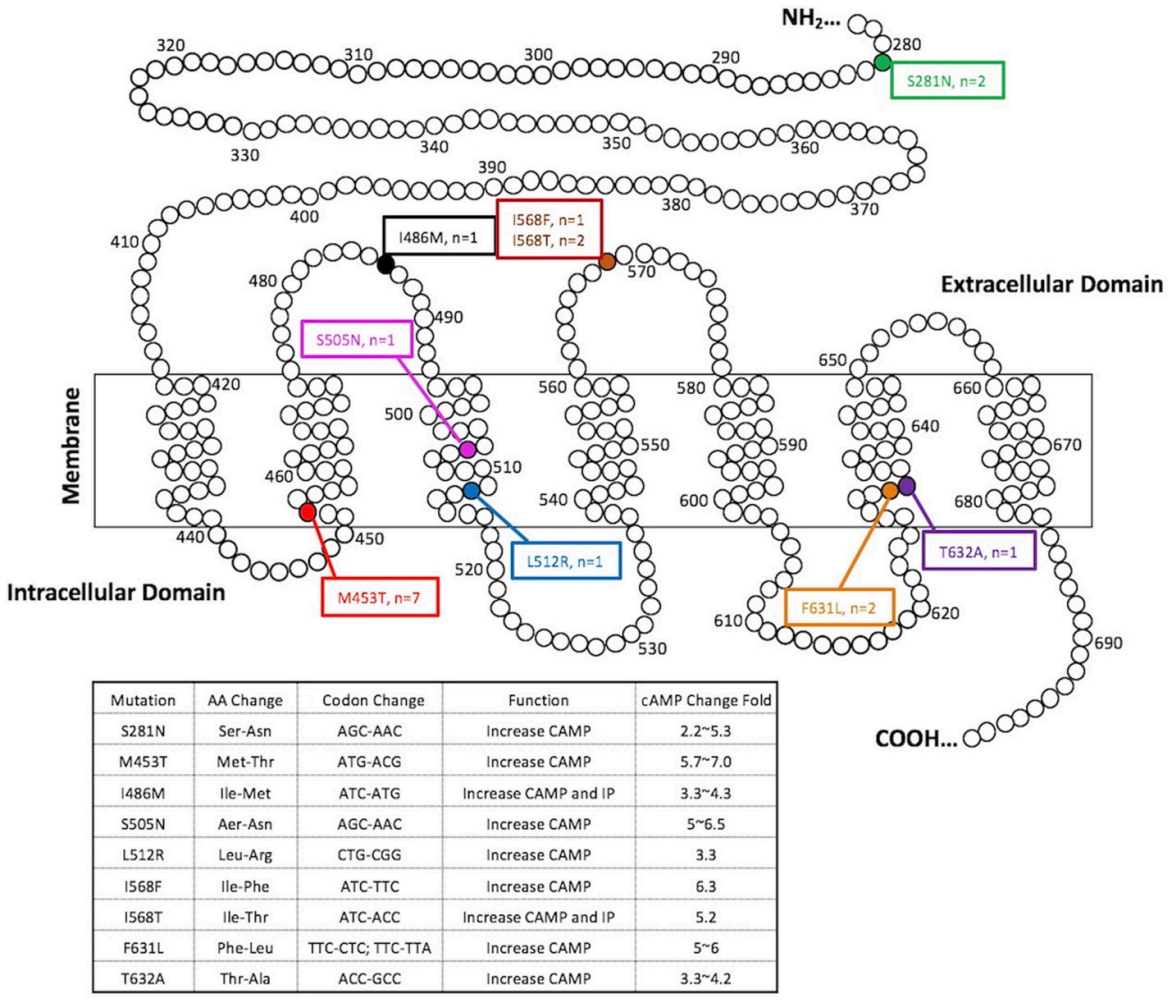
*TSHR* mutations detected in the present study. Functional characteristics of mutations are available at http://endokrinologie.uniklinikum-leipzig.de/tsh/frame.html.

Among 388 indeterminate FNBs, 136 (35.1%) were found at least one genetic alterations by the ThyroSeqV.2 next-generation sequencing panel, including gene mutations involving *BRAF, H/N/K-RAS, EIF1AX, PTEN, TERT, TP53*, and *TSHR*, gene fusions involving *IGF2BP3, NTRK3, PPARG*, and *THADA*, as well as gene overexpression of *MET* and *NIS*. *TSHR* mutations and/or *NIS* overexpression were detected in 25 (6.4%) nodules from 24 patients. Ten nodules (2.6%) harbored *TSHR* mutations only, 7 nodules (1.8%) over-expressed *NIS* gene only, and 8 nodules (2.1%) had both molecular alterations. The *TSHR* mutations were located between codons 281–640. Specifically, S281N (*n* = 2), M453T (*n* = 7), I468M (*n* = 1), S505N (*n* = 1), L512R (*n* = 1), I568F (*n* = 1), I568T (*n* = 2), F631L (*n* = 2), and T632A (*n* = 1) were identified, with codon 453 being the most frequently affected (Figure 1 and Table 1). All the mutations are known mutations that were previously reported. They have been documented to be gain of function mutations with increased cAMP production (Figure 1). The allelic frequency of the mutated *TSHR* ranged from 6 to 36% (Table 1). One nodule (#1) with *NIS* overexpression had simultaneous *EIF1AX* and *GNAS* mutations. Two nodules (#12 and #15) carrying different *TSHR* mutations (I486M and M453T, respectively) were from the same patient.

**Table 1:** Clinical characteristics of the biopsied nodules harboring NIS overexpression and/or TSHR mutations.

**Tumor no**.	**Age**	**Sex**	**Cytology**	**Gene expression/Mutation**	**Allelic frequency**	**Past history of thyroid diseases**	**Thyroid scintigraphy**	**TFT at the biopsy**	**Evidence of AITD**	**F/U outcome (F/U length)**
**NIS OVEREXPRESSION**										
1	40	F	AUS	NIS Overexpression + EIF1AX p.R13P c.38G>C + GNAS p.Q227H c.681G>T	n/a	No	Hyperfunction	Subclinical hyperthyroidism	FNB: CT	Adenomatous nodule on surgical pathology
2	43	F	AUS	NIS Overexpression	n/a	HT, clinical hypothyroidism, stopped taking LT4	No scan	Clinical hypothyroidism	History + Pathology: CT	Hashimoto's thyroiditis on surgical pathology
3	38	F	AUS	NIS Overexpression	n/a	No	Hypofunction	Subclinical hyperthyroidism	Radiology: GD	Decreased in size (12 mon), TFT normalized spontaneously
4	24	F	Suspicious-IV	NIS Overexpression	n/a	No	No scan	Euthyroid	No	Hurthle cell adenoma (7cm) on surgical pathology
5	44	F	AUS	NIS Overexpression	n/a	No	Hypofunction	Clinical hyperthyroidism	TSI: GD	Thyrotoxicosis ameliorated on MMI
6	54	F	AUS	NIS Overexpression	n/a	No	No scan	Euthyroid	No	n/a
7	38	F	FLUS	NIS Overexpression	n/a	Right benign thyroid nodules and PTMCs, post near-total thyroidectomy, stopped taking LT4	No scan	Clinical hypothyroidism	History + FNB: CT	Stable in size (5 mon)
**TSHR MUTATION AND NIS OVEREXPRESSION**										
8	31	F	FLUS	TSHR (p.M453T, c.1358T>C) + NIS Overexpression	8%	Nontoxic uninodular goiter, HT, clinical hypothyroidism, on LT4	No scan	Euthyroid	History: CT	n/a
9	35	F	FLUS	TSHR (p.M453T, c.1358T>C) + NIS Overexpression	8%	Subacute thyroiditis	Hyperfunction	Euthyroid	No	Stable in size (33 mon)
10	27	M	AUS	TSHR (p.S281N, c.842G>A) + NIS Overexpression	8%	No	Hypofunction	Euthyroid	FNB: CT	Stable in size (23 mon)
11	55	F	AUS	TSHR (p.T632A, c.1894A>G) + NIS Overexpression	11%	No	Hyperfunction	Euthyroid	FNB: CT	Stable in size (3 mon)
12	70	M	AUS	TSHR (p.I486M, c.1458C>G) + NIS Overexpression	12%	MNG	No scan	Euthyroid	No	Adenomatous nodule on surgical pathology
13	66	F	AUS	TSHR (p.L512R, c.1535T>G) + NIS Overexpression	13%	No	Isofunction	Hyperthyroidism	No	Stable in size (10 mon); Thyrotoxicosis ameliorated on MMI
14	71	F	FLUS	TSHR (p.I568F, c.1702A>T) + NIS Overexpression	24%	Thyroid nodule	No scan	Euthyroid	FNB: CT	n/a
15	70	M	AUS	TSHR (p.M453T, c.1358T>C) + NIS Overexpression	25%	MNG	No scan	Euthyroid	No	Adenomatous nodule on surgical pathology
**TSHR MUTATION**										
16	29	F	Suspicious-IV	TSHR (p.I568T, c.1703T>C)	6%	HT and MNG, post lobectomy, clinical hypothyroidism	No Scan	Clinical hypothyroidism	History: CT	Benign on surgical pathology
17	24	F	AUS	TSHR (p.M453T, c.1358T>C)	7%	No	No scan	Euthyroid	FNB: CT	n/a
18	35	F	AUS	TSHR (p.M453T, c1358T>C)	7%	MNG	Isofunction	Euthyroid	No	Stable in size (13 mon)
19	67	F	FLUS	TSHR (p.F631L, c.1893C>A)	7%	Goiter	Hypofunction	Subclinical hyperthyroidism	No	Thyrotoxicosis ameliorated on MMI
20	76	F	AUS	TSHR (p.I568T, c.1703T>C)	8%	Hyperthyroidism and MNG, took MMI 5mg/d in 2014	Isofunction	Hyperthyroidism	Radiology + TPOAb: CT	Thyrotoxicosis ameliorated on MMI
21	58	F	AUS	TSHR (p.S281N, c.842G>A)	10%	No	No scan	Euthyroid	No	n/a
22	56	F	FLUS	TSHR (p.F631L, c.1893TC>G)	11%	No	No scan	Euthyroid	No	n/a
23	61	F	FLUS	TSHR (p.M453T, c.1358T>C)	11%	MNG	Hypofunction	Subclinical hyperthyroidism	Radiology: GD and HT	Adenomatous nodule on surgical pathology
24	43	F	FLUS	TSHR (p.M453T, c.1358T>C)	11%	No	No scan	Euthyroid	No	Decreased in size (5 mon)
25	75	F	FLUS	TSHR (p.S505N, c.1514G>A)	36%	PTC, post lobectomy, subclinical hyperthyroidism on LT4 50 μg/d	Hyperfunction	Subclinical hyperthyroidism	No	Decreased in size (19 mon) after RAI, with resolution of the thyrotoxicosis

*AITD, autoimmune thyroid disease; AUS, atypia of undetermined significance; CT, chronic thyroiditis; FLUS, follicular lesion of undetermined significance; FNB, fine needle biopsy; F/U, follow-up; GD, Graves' disease; HC, Hurthle cell neoplasm; HT, Hashimoto's thyroiditis; MMI, Methimazole; MNG, multi-nodular goiter; n/a, not available; NIS, sodium-iodine symporter; PTC, papillary thyroid cancer; LT4, levothyroxine; mon, month; PTMC, papillary thyroid microcarcinoma; TFT, thyroid function test; TSHR, TSH receptor; TSI, thyroid stimulating immunoglobulin*.

### Sex and age of patients with nodules harboring *TSHR* mutations and/or *NIS* overexpression

Overall, 21 of 24 (87.5%) patients who had indeterminate nodules with *TSHR* mutations and/or *NIS* overexpression were female. All the 3 male patients in this case series presented both a *TSHR* mutation and *NIS* overexpression. Mean age of the 24 patients was (48.3 ± 16.9) years. Although difference did not show statistical significance, patients who only overexpressed *NIS* were ~10 years younger than those harboring *TSHR* mutations with or without *NIS* overexpression (mean age: 40.1 years vs. 51.7 years, *P* = 0.13).

### Sonographic patterns of nodules harboring *TSHR* mutations and/or *NIS* overexpression

Sonographic patterns of the 25 nodules with *TSHR* mutations and/or *NIS* overexpression are shown in Table 2. Of these indeterminate nodules, 13 (52%) grew in a multinodular goiter (MNG) background. Nodules harboring *TSHR* mutations with/without *NIS* overexpression had underlying MNG more commonly than nodules expressing NIS overexpression only (12/18 vs. 1/7, *P* = 0.03). The greatest dimension of the nodule on ultrasound varied between 1.0 and 5.8 cm. The mean size of nodules harboring *TSHR* mutations with/without *NIS* overexpression were smaller than nodules expressing *NIS* overexpression only (2.27 ± 1.08 vs. 3.31 ± 1.34 cm). The difference was very close to statistical significance (*P* = 0.055). Thirteen nodules demonstrated hypoechoic echotexture on ultrasound; 7 nodules were isoechoic, and the remaining 3 nodules with associated records available were of heterogeneous echogenicity. Around two thirds (17/24) of nodules were solitary thyroid nodules, whereas the remaining 8 displayed complex composition (*n* = 6) or spongiform (*n* = 2) compositions. Worrisome high-risk ultrasound features including microcalcificaion, microlobulated margin, tall shape, and suspicious neck nodes were uncommon in these nodules, shown in 3, 1, 1, and 0 nodules, respectively. Most nodules (19/23) with *TSHR* mutations and/or *NIS* overexpression had increased intranodular vascularity. Echogenicity, composition, high risk features and vascularity of the nodule did not correlate with the type of genetic changes the nodule was harboring.

**Table 2:** Sonographic patterns of the biopsied nodules harboring NIS overexpression and/or TSHR mutations.

**Tumor no**.	**Age**	**Sex**	**Cytology**	**Allelic frequency**	**MNG on US**	**Greatest dimension (cm)**	**Echogenicity**	**Composition**	**Calcification**	**Margin**	**Tall Shape**	**Suspicious nodes**	**Vascularity**
**NIS OVEREXPRESSION**													
1	40	M	AUS	n/a	Yes	2.7	Isoechoic	Complex	Microcalcification	Regular	No	No	Grade 3
2	43	F	AUS	n/a	No	4.0	Hypoechoic	Solid	No	Ill defined	No	No	Grade 1
3	38	F	AUS	n/a	No	2.7	Hypoechoic	Solid	No	Regular	No	No	Grade 2
4	24	F	Suspicious-IV	n/a	No	5.8	Isoechoic	Solid	No	Regular	No	No	Grade 4
5	44	M	AUS	n/a	No	3.3	Isoechoic	Solid	Macrocalcification	Regular	No	No	Grade 3
6	54	F	AUS	n/a	No	3.2	Heterogeneous	Complex	No	Regular	No	No	Grade 3
7	38	F	FLUS	n/a	No	1.5	Hypoechoic	Solid	No	Regular	Yes	No	Grade 3/4
**TSHR MUTATION and NIS OVEREXPRESSION**													
8	31	F	FLUS(HC)	8	No	1.2	Hypoechoic	Spongiform	No	Regular	No	No	Grade 3/4
9	35	F	FLUS(HC)	8	No	2.0	Hypoechoic	Solid	No	Regular	No	No	Grade 2/3
10	27	M	AUS	8	Yes	1.4	Hypoechoic	Complex	No	Microlobulated	No	No	Grade 3
11	55	F	AUS	11	Yes	2.0	Isoechoic	Solid	No	Regular	No	No	n/a
12	70	M	AUS	12	Yes	5.2	Isoechoic	Solid	Macrocalcification	Regular	No	No	Grade 2/3
13	66	F	AUS	13	Yes	2.2	Isoechoic	Solid	No	Regular	No	No	Grade 2
14	71	F	FLUS	24	No	1.5	Heterogeneous	Complex	n/a	n/a	Yes	No	Grade 3
15	70	M	AUS	25	Yes	3.5	Hypoechoic	Solid	No	Regular	No	No	Grade 4
**TSHR MUTATION**													
16	29	F	Suspicious-IV	6	Yes	2.0	n/a	Complex	Microcalcification and discontinuous calcifications	n/a	No	No	n/a
17	24	F	AUS	7	No	4.1	Heterogeneous	Complex	No	Regular	No	No	Grade 2/3
18	35	F	AUS	7	Yes	1.0	Isoechoic	Spongiform	No	Regular	No	No	Grade 3
19	67	F	FLUS	7	Yes	2.3	Hypoechoic	Solid	No	Regular	No	No	Grade 4
20	76	F	AUS	8	Yes	2.3	Hypoechoic	Solid	No	Regular	No	No	Grade 2/3
21	58	F	AUS	10	Yes	1.4	Hypoechoic	Solid	No	Regular	No	No	Grade 3/4
22	56	F	FLUS	11	No	2.7	n/a	Solid	n/a	n/a	n/a	No	Grade 3/4
23	61	F	FLUS	11	Yes	1.2	Hypoechoic	Solid	No	Regular	No	No	Grade 3
24	43	F	FLUS	11	No	2.6	Hypoechoic	Solid	No	Regular	No	No	Grade 2
25	75	F	FLUS(HC)	36	Yes	2.3	Hypoechoic	Solid	Microcalcifiation	Regular	No	No	Grade 3

*AUS, atypia of undetermined significance; FLUS, follicular lesion of undetermined significance; HC, Hurthle cell neoplasm; n/a, not available*.

### Functioning status of nodules harboring *TSHR* mutations and/or *NIS* overexpression

Among 25 nodules with *TSHR* mutations and/or *NIS* overexpression, 12 (48.0%) had NM thyroid update and scan checked, 5 of which were done between 21 days and 3 years before the FNB and otherwise after the FNB. Four of them exhibited hyperfunctioning nodules on thyroid scintigraphy, while 5 were hypofunctioning, and 3 were isofunctioning nodules compared to surrounding normal thyroid parenchyma. Accumulation of radioiodine by the nodule did not correlate with the type of genetic changes in the target nodule.

### Biochemical thyroid function and co-existing autoimmune thyroid disease (AITD) in patients with nodules harboring *TSHR* mutations and/or *NIS* overexpression

**Figure 2:**
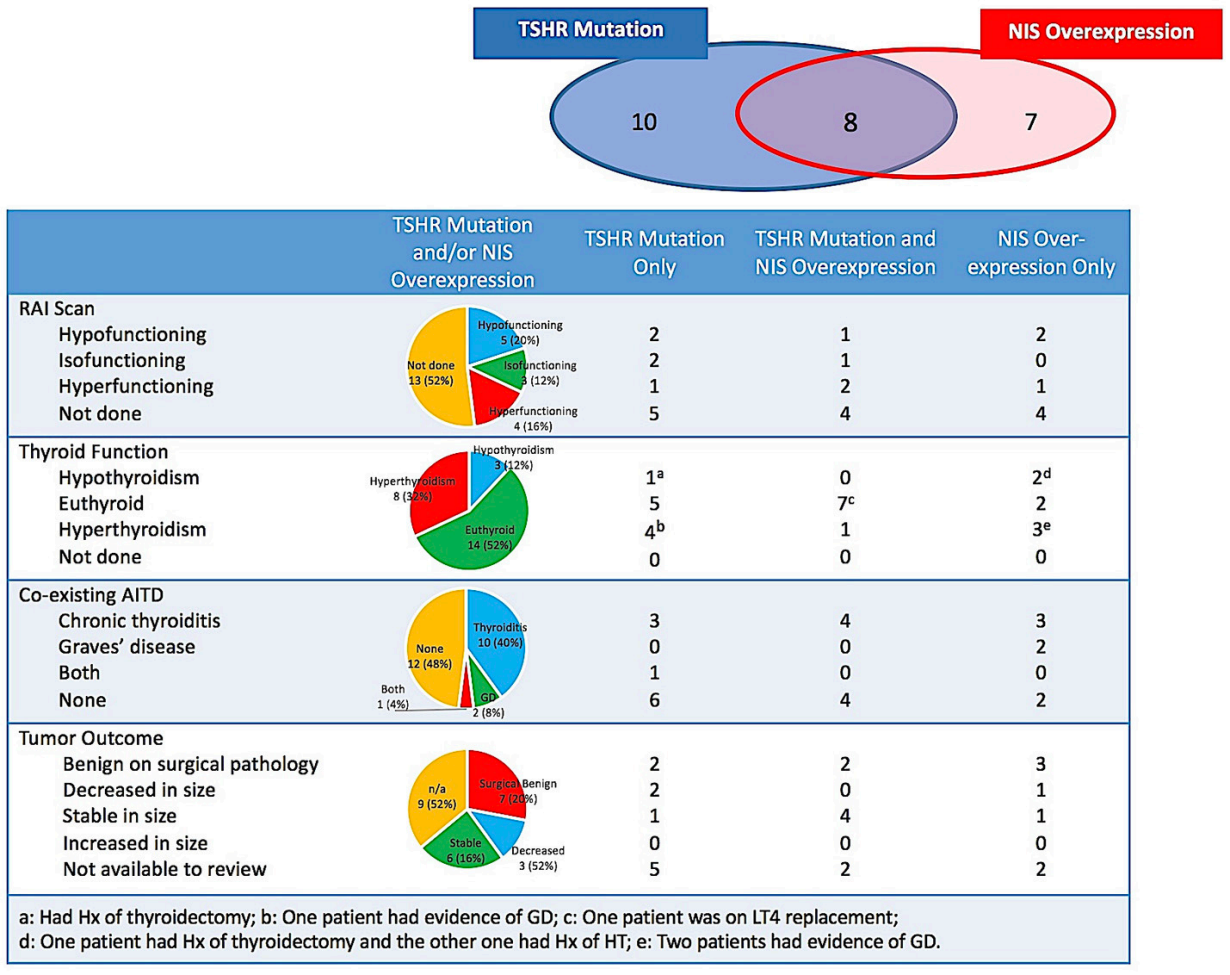
Accumulation of radioiodine, biochemical thyroid function, co-existing AITD and tumor outcome of the biopsied nodules harboring *TSHR* mutations and/or NIS overexpression. AITD, autoimmune thyroid disease; GD, Graves' disease; HT, Hashimoto's thyroiditis; Hx, history; NIS, sodium-iodine symporter; LT4, levothyroxine; RAI, radioiodine; TSHR, TSH receptor.

Biochemical thyroid function tests were available for review in 23 (95.8%) patients. None of the patients were taking thyroid medication including levothyroxine, thyroid extract, antithyroid drugs, or iodine contained preparations at the time of evaluation, except for 1 case of Graves' disease (GD) treated with Methimazole (MMI) indicated in Figure 2. Of the 24 indeterminate nodules from 23 patients, more than half (*n* = 14, 56%) existed with normal biochemical TFTs, 8 had concurrent hyperthyroidism in which only 2 were caused by the autonomous function of the biopsied nodules, and 3 had concurrent hypothyroidism as the consequence of previous surgeries or chronic thyroiditis (CT). Evidence of co-existing AITD in the target nodule and/or its surrounding thyroid parenchyma were indicated, by medical history, cytology, thyroid autoantibody measurements, ultrasound in 52% (*n* = 13) of nodules (Table 1 and Figure 2). Patients with and without AITD did not show significant difference in age and sex distribution (44.4 ± 16.7 vs. 53.0 ± 16.6, *P* = 0.11; 12:1 vs. 10:1, *P* = 0.26). Biochemical thyroid function and co-existing AITD did not correlate with the type of genetic changes in the target nodule.

### Follow-up of nodules with *TSHR* mutations and/or *NIS* overexpression

Seven nodules underwent surgeries and all were reported benign on final pathology, including one Hurthle cell adenoma, 4 adenomatous nodules, 1 benign thyroid nodule and 1 fibrous variant Hashimoto's thyroiditis. Nine nodules without surgery were followed up by ultrasound between 3 and 33 months (median 12 months): 6 nodules were stable and 3 had decreased in size, respectively. Ultrasound follow-ups were not available in the additional 9 nodules, either because of short time since biopsy (< 6 months, *n* = 2) or because of loss to ultrasound follow-up at the Boston Medical Center. Thus, *TSHR* mutations and *NIS* overexpression have not shown any solid association with the malignancy in our patient cohort.

### Featured cases

It is worthwhile to highlight three cases. The first case is a 40-year female. Testing of her nodule detected *NIS* overexpression, as well as simultaneous *EIF1AX* and *GNAS* mutation. This is the only case with multiple gene mutations in our cohort. The nodule showed microcalcification and otherwise no suspicious features on ultrasound. It manifested as hyperfunctioning on the NM scan and a low TSH level consistent with subclinical hyperthyroidism. The patient decided to be put on medical therapy since August, 2017. Her TFT returned to normal after taking MMI 5–10 mg daily for 2 months and the nodule did not develop suspicious sonographic features or grew significantly (>20% in 2 dimensions) on periodic ultrasound examination. The second case is a 70-year male originally from Vietnam, a country with mild iodine deficiency (http://www.ign.org/vietnam.htm). In a background of big MNG, 2 nodules were identified with indeterminate FNBs. Both nodules had *TSHR* mutations, but the codons affected were different (p.I468M and p.M453T, respectively). The patient underwent a total thyroidectomy by a high-volume surgeon and surgical pathology confirmed that both nodules were adenomatous nodules. A papillary microcarcinoma (0.5 cm, conventional subtype, BRAF V600E mutation absent) and a 0.3 cm non-invasive follicular neoplasm with papillary-like nuclear features (NIFTP) loci were incidentally found in the contralateral thyroid lobe according to the post-surgical pathology report. Levothyroxine was given to keep his TSH low-normal level after surgery. Seven months after the total-thyroidectomy, ultrasound monitoring indicated thyroid tissue remnants in both thyroid beds measuring (2.8 × 1.1 × 1.1) cm and (0.7 × 0.5 × 0.8) cm, respectively. The third case is a 75-year female with a hot nodule harboring TSHR p.S505N mutation in high allelic frequency (36%). She presented with subclinical hyperthyroidism due to the hot nodule, without any thyroid active medication. Her past medical history included a lobectomy for MNG and papillary thyroid carcinoma more than 30 years ago. Although complete thyroidectomy was recommended, she refused for the concern about risks of reoperation. She was treated with radioiodine in January, 2017 and 3 months later, her nodule decreased in size with resolution of the thyrotoxicosis.

## Discussion

In the present study, we have investigated the clinical significance and diagnostic utility of *TSHR* mutations and/or *NIS* overexpression in thyroid FNB specimens with indeterminate cytology. Unlike previous studies which checked somatic genetic alterations of *TSHR* and *NIS* using surgically resected thyroid tissue from known autonomous nodules, the distinction of our study was having these molecular alterations detected in pre-operative thyroid nodules with indeterminate biopsy. Accordingly, the purpose of detecting *TSHR* mutations and/or *NIS* overexpression in thyroid nodules was different between our study and former researches. Instead of verifying the etiological involvement of *TSHR* and *NIS* alterations in autonomously hyperfunctioning nodules, we determined in a clinical utility study the true prevalence of TSHR mutations and NIS overexpression with patients with indeterminate thyroid cytology.

Our study demonstrated that *TSHR* mutations and/or *NIS* overexpression occurred in a higher than expected 6.4% of 388 consecutively biopsied thyroid nodules with indeterminate cytology. In regards to the prevalence of *TSHR* mutations in this nodule series, it was 4.7%. All the *TSHR* mutations are known mutations that were previously reported in AFTNs, and are somatic gain-of-function mutations (Figure 1) (36–39). In previous studies, somatic mutations of *TSHR* gene have been reported in toxic adenomas and toxic multinodular goiters, with a frequency ranging from 8 to 82% of cases (40, 41). It is not surprising that the mutation rate described in our study was much lower when compared with existing data, because we were not doing the molecular testing in selected functioning nodular diseases. Interestingly, the most frequently detected *TSH* mutation in our FNB series was M453T, which is also the most prevalent mutation in tissue samples from known AFTNs (42). It indicated that this mutation would account for a major genetic alteration of *TSHR* in thyroid nodules and be more common than we used to anticipate. Given the fact that gain-of-function mutations of the *TSHR* can constitutively stimulate TSH-adenylcyclase-cAMP pathway to increase the uptake of iodine by thyrocytes and the production of thyroid hormones (4), we expected that *NIS* overexpression would commonly happen along with *TSHR* mutations. However, more than half (10/18) of *TSHR* mutations were not accompanied with over-expressed *NIS* gene. Type and allelic frequency of the *TSHR* mutation did not influence the occurrence of *NIS* overexpression. On the other hand, we found 7 nodules having *NIS* overexpression without any *TSHR* mutations. A proposed explanation may relate to other known or unknown molecular alterations and alternate cellular pathways besides TSHR that are mediating the increase in *NIS* expression. The nodule that overexpressed *NIS* had concurrent *EIF1AX* and *GNAS* mutations found in our study was a clue for this hypothesis. Iodine deficiency may be another influence on the occurrence of *TSHR* mutations and *NIS* overexpression in our study. Previous studies have suggested that AFTNs and toxic nodules in a MNG in iodine-deficient regions tend to have an elevated frequencies of *TSHR* mutations (43–45). Iodine status is a major factor that affects the expression of *NIS* gene. *NIS* expression in thyrocytes is elevated when iodine is deficient and this in turn retains the ability to accumulate iodine to maintain thyroid hormone production; while iodine excess down-regulates NIS expression (46). However, a study done in Turkey did not find that *TSHR* mutation was influenced by iodine intake (16), and the mutant *TSHR* was a common genetic cause of TA in Japanese, despite their high iodine intake (42). The effect of iodine intake on genetic alterations of *TSHR* and *NIS* remains unclear and attractive. Unfortunately, in the present study, former and current iodine intakes of each case was not available for further analysis.

For many years, AFTNs have been considered a very low risk for malignancy and FNB of such nodules is therefore normally unnecessary. The discovery of *TSHR* mutations and *NIS* overexpression (26, 47) in AFTNs not only supports an important molecular mechanism underlying the pathogenesis of non-autoimmune thyroid autonomy, but also encourages the idea that *TSHR* and *NIS* alterations can be used as molecular markers to predict AFTNs and subsequently reduce FNBs on them. On the other hand, although genetic analysis of the *TSHR* gene in thyroid cancers supported the notion that *TSHR* mutations do not play a role in the pathogenesis of nonfunctioning differentiated thyroid carcinoma (48), as TSHR mediates the proliferation of thyrocytes through the TSH-AC-cAMP pathway, we cannot rule out that its constant activation by a gain-of-function mutation is a logical carcinogenic factor (48, 49). Based on prior reported cases and experimental studies (25, 49), *TSHR* mutations, especially those occurring with a high allelic frequency (>30%) and/or at specific codons (e.g., between 620 and 631), are associated with the uncommon but relatively increased risk of functioning thyroid carcinomas. Therefore, we should precisely evaluate the predictive value of *TSHR* mutations for AFTNs and malignancies according to more detailed mutation information, e.g., type, allelic frequency, other co-existing mutation, etc. The NGS approach to *TSHR* mutations and *NIS* expression detection in thyroid FNB specimens with indeterminate cytology allows us for the first time to correlate details of these genetic alterations with functional status and outcome of the target thyroid nodules. This gives us a better understanding of the clinical significance of using *TSHR* mutations and *NIS* overexpression as molecular makers. In our study, out of 25 nodules harboring mutant *TSHR* and/or *NIS* overexpression, radioiodine scintigraphy was performed in 12 and TFT results were available in 24. Surprisingly, these data indicated that accumulation of radioiodine by the nodule and biochemical thyroid function of the resulting phenotype were quite variable, and did not correlate the TSHR mutation and NIS overexpression in the target nodule. Thus, *TSHR* mutations and *NIS* overexpression detected in pre-operative FNBs are not good molecular indicators of hyperfunctioning nodules. But we observed that nodules with multiple genetic alterations (*NIS* overexpression, *EIF1AX*, and *GNAS* mutations) and mutant *TSHR* with a high allelic frequency (36%) were both hyperfunctioning. It likely suggests that co-existence of other mutations and abundant mutant *TSHR* may be necessary to attain enough activation and induce autonomous function in the target nodule. In regards to the value of *TSHR* mutations and/or *NIS* overexpression in distinguishing benign and malignant nodules, based on surgical pathology and ultrasound follow-up, these genetic alterations did not predict any malignant diagnoses. This result is in accordance with our expectation, given that *NIS* expression represents well-differentiated status of thyrocytes, and only one case of a *TSHR* mutation with high allelic frequency was detected. We agree with the current opinion that *TSHR* mutations (except for those found at a high level) and/or *NIS* overexpression do not raise the probability of thyroid cancer or a pre-cancerous tumor, noninvasive follicular thyroid neoplasm with papillary-like nuclear features (NIFTP).

Previous studies showed that activating *TSHR* mutations only existed in clinical and histological subtypes of autonomous nodules, but had never been found in non-functioning nodules. It has been also reported that cold nodules express very low levels of *NIS* mRNA (26). However, the results of our study directly contradict the former finding. Nodules harboring mutant *TSHR* associated with elevated cAMP production and/or *NIS* overexpression could display hyperfunction, isofunction, or even hypofunction, on thyroid scintigraphy. Taking into account that the allelic frequency of *TSHR* mutations in the majority of these nodules was relatively low (between 7 and 13%), our finding may suggest that autonomous function requires higher allelic frequency of the mutant *TSHR* levels; on the other hand, a negative finding in non-functioning nodules from previous studies may be due to less sensitive methods they employed for detecting these genetic alterations compared to the highly sensitive NGS used in this study.

Our study also illustrated for the first time that co-existing AITD was common in patients with a nodule harboring *TSHR* mutations and/or *NIS* overexpression. Although more than half of the patients were found with evidence of AITD, i.e., Graves' disease and/or chronic autoimmune thyroiditis, 71% (5/7) with NIS overexpression demonstrated AITD. This figure is much higher than the prevalence of AITD in the general population (50). Mechanisms underlying this phenomenon are not clear. Certainly, high thyroidal radioactive iodine uptake is seen in early Hashimoto's thyroiditis but usually under the condition of an elevated TSH and TSHR activation. Theoretically, modifications in the primary structure of the *TSHR* might be an autoimmune antigen causing an immunoreaction. However, a couple of somatic *TSHR* variants that had been proposed as the cause of autoimmunity, including Asp36His and Pro52Thr, were later demonstrated to be polymorphisms that were also frequently present in healthy people (51, 52). It would be interesting to explore the possibility that variant TSHR, when associated with particular HLA haplotype(s) or other genetically linked anomalies in the immune system can grant higher susceptibility to developing AITD. Other than mutant *TSHR*, co-existing AITD may not result from *NIS* overexpression, but is more likely one of factors that causes elevated NIS expression. Nonetheless, we should realize that AITD, when concurrent with genetic alterations, is a confounding factor on the phenotype of biochemical thyroid function and ultrasound sonography. For example, Graves disease plus a functioning nodule with a *TSHR* mutation may cause more apparent hyperthyroidism, while Hashimoto thyroiditis may mask the hyperthyroidism caused by the nodule harboring a gain-of-function *TSHR* mutation. This can be one explanation to why some autonomous functioning nodules present in patients with normal thyroid function.

Though our study provided a novel perspective to understand the clinical significance of *TSHR* mutations and/or *NIS* overexpression in thyroid nodules, due to some limitations, results of this study need to be applied with caution. First, as we mentioned above, iodine intake and iodine nutrition status of these cases were not available. Thus, the influence of iodine on these genetic alterations could not be analyzed but NHANES studies have seen a stable median iodine intake based on urinary iodine has been adequate in the United States for >30 years (NHAS references). Secondly, only some patients performed thyroid scintigraphy and most patients did not undergo surgery, so the predictive value of these genetic alterations may be over- or under-evaluated. Thirdly, radioiodine scintigraphy was performed in planar technique. This may not be sensitive enough to define thyroid autonomy of nodules without TSH suppression, leading to potential misjudgment on function of target nodule. Finally, length of follow-up was not long enough, hence clinical development of the target nodule has not been fully exposed.

In conclusion, *TSHR* mutations and/or *NIS* overexpression can be detected in pre-operative FNB specimens using the NGS approach. These genetic alterations occurred in 6.4% thyroid nodules in this consecutive series with indeterminate cytology. One third of these nodules had both a *TSHR* mutation and *NIS* overexpression. Co-existing AITD was common in cases with these molecular alterations. Thyroid function and accumulation of radioiodine varied and did not correlate with these genetic changes in indeterminate nodules. None of our patients with *TSHR* mutations and/or *NIS* overexpression manifested malignant outcomes. How to use these two molecular markers in thyroid FNBs to guide our clinical practice warrants further investigation.

## Author contributions

SL designed the study. HG, DM, and GT reviewed clinical records, collected data, and did data input. HG prepared the manuscript, tables and figures. SL revised the manuscript. All authors reviewed the manuscript.

### Conflict of interest statement

The authors declare that the research was conducted in the absence of any commercial or financial relationships that could be construed as a potential conflict of interest.
